# Will COVID-19 Mark the End of an Egalitarian National Health Service?

**DOI:** 10.1017/err.2020.33

**Published:** 2020-04-09

**Authors:** Sabrina GERMAIN

**Affiliations:** * Senior Lecturer in Law, City, University of London (The City Law School), London, UK; email: sabrina.germain@city.ac.uk.

## I. Introduction

The exceptional circumstances brought about by the COVID-19 pandemic have affected the traditional organisation of healthcare resources allocation in the UK. Since its inception, the National Health Service (NHS) has aimed to regulate risks of ill health in the population by providing an equal and universal provision of healthcare services to residents based on their health status rather than their ability to pay. The rapid spread of this new virus has, however, triggered a shift in paradigm from an egalitarian allocation of healthcare resources to a utilitarian approach, which has led to discussions about society’s greatest taboos: death and dying and the economic value of individuals’ health.

The rapid growth of COVID-19 cases around the world has also highlighted the difficulties governments have had in dealing with the allocation of scarce resources. Even though the NHS remains publicly funded, the provision of services is now ranking the needs of patients that are directly or indirectly affected by the virus rather than providing equal access to treatment for all. This paper argues that the current government’s emergency healthcare policy has thereby favoured a utilitarian approach to healthcare rationing and potentially initiated the end of an egalitarian NHS.

The paper first unpacks why the allocation of healthcare resources is fundamentally a question of justice in Britain and explains why healthcare law and policy require a philosophical approach in times of crucial change and crisis. Secondly, the paper provides a critical analysis of the current situation for the allocation of healthcare resources and the provision of services to patients directly or indirectly affected by the virus. It concludes that the liberal egalitarian conception of distributive justice at the heart of the NHS that aims to guarantee free and equal access to healthcare is now in jeopardy and is being replaced by a utilitarian approach based on a priority ranking of patients for the provision of services at this critical time.

## II. Justice at the heart of the British healthcare system

The resources available and mobilised for healthcare in the UK have been out of sync with the growing needs of society long before the surge of COVID-19.^1^ In fact, the scarcity of these resources has mandated patterns for their allocation ever since the inception of the NHS.^2^ Aneurin Bevan, founder of the NHS, had established that healthcare resources had to be available universally based on patients’ needs rather than their ability to pay, in order to stop ill health in the population after the war. This automatically placed the national institution within a framework of justice.^3^ Nonetheless, considerations around the basic entitlement to healthcare, whether resources should be allocated based on a patient’s, a community’s or a population’s needs or whether the NHS should aim to provide individuals with greater life opportunity by satisfying healthcare needs, still to this day occupy the policy debate, and most particularly in this time of crisis.

In theory, justice mandates that we treat equally those who are alike and those who are different in proportion to their differences. Justice balances the needs and desires of individuals with the claims of the community.^4^ It is concerned with human relationships in the social order and issues of distribution. Distributive justice, as an element of justice, provides methods for the allocation of resources. Moral political philosophers have called on different ideas of distributive justice such as liberal equality, utilitarianism, communitarianism and libertarianism to create appropriate allocation frameworks for healthcare.^5^ Each of these four conceptions of distributive justice can form the basis of a healthcare policy. At times, they are also merged to adjust the distribution of healthcare resources.

Granted, healthcare resources do not possess outstanding attributes in comparison to other health determinants. However, their moral significance derives from the role they play in our lives. The pattern chosen for their allocation must therefore focus on the attainment of justice.^6^ It is the indisputability and seriousness of healthcare needs that make the distribution of these resources stand out from the allocation of any other consumer good. Their importance stems from the potential they have to alleviate risk of illness, suffering and absolute harm.^7^

In line with these theoretical considerations, the bedrock of liberal egalitarian justice on which rests the NHS aims to provide equal access to care through the availability of publicly financed services at the point of use.^8^ This has fostered a sense of pride in the British population and explains in part why over the past 70 years the egalitarian core of the NHS has been adapted but has persisted in spite of major political and economic shifts.^9^ Even though crucial turning points at the national level have triggered healthcare reforms that embraced alternative ideas of justice, at times prescribing the use of utilitarian means or libertarian principles to achieve greater efficiency and guarantee equal access to care, the original egalitarian goals have not been compromised.^10^

After the war, Bevan was convinced that “illness should neither be an indulgence for which people have to pay, nor an offence for which they should be penalised, but a misfortune, the cost of which should be shared by the community”.^11^ There was also the necessity to respond to fear with a collective action led by the state. From the start, the NHS rested on ideas of fairness and equality and an understanding that resources had to be shared in order for the rich and the poor to have the same access to services.^12^ All would be treated equally, even the most vulnerable patients, thanks to the redistribution of resources and a system of provision based on needs rather than means. The NHS was to level the healthcare “playing field” by providing more care to the least favoured and most vulnerable, as well as equal access to services for all other types of patients.^13^

Over the years, successive governments have had to reinterpret Bevan’s commitment because of diminishing resources and growing healthcare needs. The rationing of resources became an underlying theme in healthcare policy as early as the 1980s. The change in culture embracing libertarian and utilitarian methods in healthcare that was initiated at the beginning of the Thatcher era^14^ was taken forward by New Labour in the 1990s and climaxed under the coalition government. The NHS principles of equality in healthcare were preserved, but a neoliberal approach started to be adopted for the delivery of healthcare services.^15^

This historical evolution of ideas of justice in British healthcare is of interest when assessing the impact of changes brought by times of crisis. After Brexit,^16^ COVID-19 is yet another crucial turning point for the NHS policy-makers having to react in urgency to a situation that may require them to leave behind its egalitarian foundations to embrace a more utilitarian approach to the provision of healthcare services.

## III. COVID-19: a paradigm shift

The current pandemic confronts the NHS with unprecedented demands and pressures with a direct effect on intensive care units and the medical workforce.^17^ Some groups of patients, such as the elderly and individuals affected by comorbidities, have recorded greater fatality rates; others affected by underlying socioeconomic inequalities that were at play prior to the spread of the infection are also more prone to contracting the virus.^18^ Nevertheless, the infection is “non-discriminatory” in nature, affecting all social statuses and affluences.^19^ This does not, however, translate into a levelling of access to healthcare resources for all.^20^ Dramatic ethical dilemmas relating to resource allocation are thereby brought into sharper focus. The government has to determine how to allocate scarcer resources with the growing and urgent need to mitigate the impact on the NHS and the population.

If the British healthcare system were to keep in line with its principles of egalitarian justice, healthcare services would be delivered equally to all similarly situated patients on the territory. This would entail that patients be treated alike regardless of whether or not they have contracted the virus. Additional resources would, however, be provided to more vulnerable or critical patients in comparison to less urgent cases as an exception to this strict equality rule.^21^ However, the reality of the pandemic does not allow for the system to spread out its resources in order to preserve equality. For instance, patients cannot be rotated to share ventilator time or bed days in hospital. The system therefore automatically reverts to a de facto “first come, first served basis” until resources are depleted.^22^ In the near future, a critical juncture will be reached where clinicians will be forced to choose between providing life-sustaining conditions to patients or abandoning treatment.

Such concrete discussions around death and the cost associated with life-sustaining treatments have never been so pressing, but the race against the virus no longer allows for a pause to reflect on a collective decision around the allocation of healthcare resources in times of crisis. Thus, the government, along with other European counterparts, is operating a shift in healthcare policy towards a utilitarian model of rationing with equality in access as a second-rank priority. The aim is to maximise healthcare outcomes by favouring individuals with a greater chance of survival and introducing a ranking of patients.^23^

For instance, on 21 March 2020, the National Institute for Health and Care Excellence (NICE) has introduced, at breakneck speed, its COVID-19 rapid guidelines to facilitate intensive care clinicians’ assessment of patient needing to be admitted into critical care. The recommendations suggest that doctors consider the medical benefit, including the patient’s likelihood of recovery from critical care admission, to an outcome that is acceptable.^24^ The advice requires upstream decision-making upon the patient’s admission and a certain level of speculation on how well they will respond to critical care. Only time will tell whether these criteria have indirectly imposed age-based rationing in the sense that elderly patients tend to have a greater propensity for comorbidities and may be assessed as having less of a likelihood of recovery from critical care. At a philosophical level, our society also needs to decide whether forgoing equality in access to care for the elderly is a choice that needs to be made in a time of pandemic; whether these older patients have had their “fair innings” and should sacrifice their care for younger patients more likely to recover from the infection,^25^ an approach that seems to have been taken by the current government.

Other vulnerable groups of patients also feel the repercussions of this change in allocation strategy, mostly patients suffering from chronic conditions such as diabetes or cancer. Individuals in need of living-donor transplantation have also had their surgeries postponed or cancelled for fear that they would take up intensive care beds at post-op. Routine check-up appointments have for some part moved online, but patients suffering from chronic illnesses that may require additional attention tend to refrain from asking for help for fear of overburdening the healthcare system.^26^

At the diagnostic level, utilitarian considerations are also coming into play. As the UK entered the “delay” phase of its plan to fight COVID-19 on 12 March 2020, policy shifted with regards to the testing of individuals at risk of having contracted the virus. The NHS no longer offered testing in the community and reserved the process to the hospital setting for immunocompromised and intensive care patients.^27^ Other patients with symptoms that did not require inpatient medical care were asked to self-isolate without an official diagnosis.^28^ These measures were rolled out in parallel to a letter sent to primary care doctors that set out a list of activities to be halted or postponed in GP surgeries.^29^ Equality in access to preventative medicine was thereby replaced to allocate scarce resources to individuals more at risk.

In addition, in an effort to provide a centralised response to the crisis, the government has drafted emergency legislation. Among other special stipulations, the Coronavirus Act encloses provisions to grant a temporary authorisation of practice to designated healthcare professionals and emergency volunteers.^30^ The effort to rally a greater number of medical professionals raises other ethical questions that should be addressed in light of the new utilitarian approach to resource allocation. Even though, after much pressure, the government took the decision to test healthcare workers showing symptoms, the lack of routine testing of key workers certainly endangers patients.^31^ Not aware of their health status, asymptomatic health workers may transmit the disease while providing care.^32^ It may well be that we should now consider providing preferential treatment to this essential group of workers. In times of health emergency, it is important to guarantee that the medical forces be as fit as possible and in the event that they do fall sick that they return to work swiftly for the benefit of the population. Simply put, prioritising healthcare professionals’ wellbeing and treatment can be justified because it would help maximise the health outcome of the entire population.^33^

Most striking is the emphasis put by public powers on individual responsibility and collective action. Without any available treatment or vaccine, the government sought to relieve pressure from the NHS by incentivising the public to adopt social distancing measures and for individuals to self-isolate if they suspected they have the virus.^34^ In an effort not to detract resources from the healthcare system, British society could now reflect on another conception of distributive justice that would embrace communitarian approaches to rationing. Communitarian thoughts and justice theories tend to focus on a balanced allocation of resources that takes into account a patient’s illness but also the needs of the local community to achieve just outcomes for society as a whole.^35^ If we are to think collectively about healthcare outcomes, we should also seriously account for underprivileged groups such as rough sleepers, drug users and the homeless during a pandemic. These groups are less likely to have access to the NHS as they are for most part unregistered residents and may not be able to practice daily hygiene, putting them at a greater risk of getting infected.^36^

Protection of vulnerable groups and at-risk patients is an upstream process that will require some redesign of current healthcare institutions after the pandemic. Offering appropriate healthcare support to immigrant populations, improving sanitation and access to medical services in shelters as well as increasing access to care for people with disabilities are only a few examples of initiatives that must be put into place to protect these groups from future epidemics.^37^

## IV. Conclusion

Debates around who should meet their death first make us uneasy as a society.^38^ This is in part the reason why we have missed out on an opportunity for a collective dialogue at the onset of the pandemic. We now leave some of the most tragic decisions in the hands of clinicians on the ground.^39^ Triage among incoming patients occurs on a daily basis; however, choices at a systemic level, such as the allocation of intensive care beds, ventilators and test kits among different hospitals, remain in the remit of the government. Clinicians will continue to follow deontological principles and act in the best interests of their patients. These principles may sometimes conflict or work independently from governmental guidelines that dictate the allocation of resources at a macro level.^40^ Nonetheless, clinicians will have to adjust their clinical assessment to mitigate resources and maximise the greater good in line with the government’s utilitarian healthcare policies.^41^ All of these moral decisions cannot be made in silos; healthcare workers need support from their professional colleges and colleagues from other disciplines, among which are political theorists and bioethicists, to make the best judgement calls.^42^ Indeed, the pandemic has demonstrated that distributive justice and its different conceptions (egalitarianism, utilitarianism, communitarianism or libertarianism) are no longer confined to theoretical assumptions but anchored in reality and that they must be used as a first port of call to find models for the allocation of scarce resources.

The Second World War had brought about a national egalitarian institution for the care of the British people. The current “war” on the virus has given rise to a new wave of utilitarianism for the provision of healthcare services. With a realistic outlook on the situation it is clear that the aftermath is not in close sight, with a vaccine potentially only available in 12–18 months. The cost of having scaled up critical and intensive care on the entire territory and having purchased services and hospital beds in the independent sector will have long-term effects on NHS finances. Going back to the egalitarian model that was already under strain prior to the advent of COVID-19 will be practicably impossible, even if current utilitarian emergency policies are suspended. The NHS will nonetheless need to first address the delays in treatment that occurred during the time of the pandemic. Second, the public health strategy will have to be reassessed to prepare for a potential future incident of a similar scale and to learn the lessons from the current episode.

It is now time for us to accept that the NHS will face hard choices in the weeks, months and even years to come as a consequence of this devastating pandemic. Unfortunately, some will be direct and others indirect victims of COVID-19, for lack of critical and intensive care resources or because of delays in treatment. We should, however, remind ourselves that resources continue to be used to save as many lives as possible in order to offer all a fair and equal opportunity to pursue life plans upon recovery from an illness.^43^ Things may never go back to the way we knew them before this pandemic, especially with regards to the healthcare system and resources available. We are entering a new period that has triggered personal and collective grief for this loss of normalcy.^44^ This overhaul may, however, have given us an opportunity to discuss as a society the resources we wish to allocate to the NHS in the future and the manner in which we believe it is most just to ration what is now available.

## Footnotes

1 See, generally, DJ Hunter , The Health Debate (Bristol, 2nd edn, Policy Press 2016).

2 D Weinstock, “Healthcare in Political Philosophy: What Kind of a Good Is It?” (Research Centre on Ethics, University of Montréal, 2010) p 1.

3

TL Beauchamp and JF Childress , Principles of Biomedical Ethics (Oxford, Oxford University Press 2001) p 243 .

4 See, generally, HH Joachim and DA Rees (eds), Aristotle: The Nicomachean Ethics (Oxford, Clarendon Press 1953).

5

S Germain , Justice and Profit in Health Care Law: A Comparative Analysis of the United States and the United Kingdom (London, Hart Publishing 2019) pp 15–45 .

6

S Segall , “Is Health Care (Still) Special?” (2007) 15(3) The Journal of Political Philosophy 342 .

7

T Schramme , “On Norman Daniel’s Interpretation of the Moral Significance of Healthcare” (2009) 35(1) Journal of Medical Ethics 17 1910393710.1136/jme.2008.024208.

8

C Newdick , “Promoting Access and Equity in Health: Assessing the National Health Service in England” in CM Flood and A Gross (eds), The Right to Health at the Public/Private Divide: A Global Comparative Study (Cambridge, Cambridge University Press 2014) p 107 .

9
ibid.

10 Germain, supra, note 5, 140–73.

11 Aneurin Bevan, Minister for Health 1945–1951.

12 Newdick, supra, note 8, 107.

13

WH Beveridge , Social Insurance and Allied Services (London, HM Stationary Office 1942 PMC256077510916922). This model echoes John Rawls’ theory of justice. See, generally, J Rawls , A Theory of Justice (Cambridge, MA, Harvard University Press 2005 ).

14 In 1979, the Thatcher government introduced competition and efficiency in social policy to fight overwhelming public expenditures. After a controversial review of the NHS, the Conservatives introduced the purchaser–provider divide in healthcare. Universality of care was preserved and equality in access was upheld, but resources simply had to be optimised to derive the greatest utility from a more “efficient” NHS in order to address the needs of vulnerable patients. See National Health Service and Community Care Act 1990.

15 The Health and Social Care Act (2012) placed the patient at the heart of the system and medical professionals in a support role. The NHS principles of equality in healthcare were preserved, but a neoliberal approach to the delivery of healthcare services had been adopted. Healthcare had become a fully fleshed market where the private sector was encouraged to compete with providers to offer the best and most cost-efficient services to patients. These changes shaped the place given to the independent sector (private and corporate entities) in the financing and provision of healthcare services in the twenty-first century.

16

S Germain , “Will There Be Justice in Healthcare Post-Brexit?” in E Fahey and T Ahmed (eds) On Brexit: Law, Justices and Injustice (Cheltenham, Edward Elgar 2019) pp 158–74.

17

M Morgan , “When the Problem Is Urgent and Important” (2020) BMJ 10.1136/bmj.m106232184226 <https://www.bmj.com/content/368/bmj.m1062> (last accessed 31 March 2020).

18 See, generally, A Resnick , S Galea and K Sivashanker , “Covid-19: The Painful Price of Ignoring Health Inequalities” (2020) BMJ <https://blogs.bmj.com/bmj/2020/03/18/covid-19-the-painful-price-of-ignoring-health-inequities> (last accessed 31 March 2020).

19 The British Prime Minister Boris Johnson and his Health Secretary Matt Handcock have contracted the virus, proving that even the highest echelons of power could not be sheltered from this infection.

20 Resnick, Galea and Sivashanker, supra, note 18.

21 The pioneering work of John Rawls on liberal equality establishes that justice requires that all be treated equally and that inequalities be repaired with a system of distribution based on morally relevant factors. Thus, according to Rawls’ difference principle, inequalities are permissible as long as they provide the greatest benefit to the least advantaged. See Rawls, supra, note 13.

22

C Pagel , M Utley and S Ray , “Covid-19: How to Triage Effectively in a Pandemic” (2020) BMJ <https://blogs.bmj.com/bmj/2020/03/09/covid-19-triage-in-a-pandemic-is-even-thornier-than-you-might-think> (last accessed 24 March 2020).

23

D Shaw , D Harvey and D Gardiner , “Don’t Let the Ethics of Despair Infect the Intensive Care Unit” (2020) Journal of Medical Ethics <https://blogs.bmj.com/medical-ethics/2020/03/26/dont-let-the-ethics-of-despair-infect-the-intensive-care-unit> (last accessed 27 March 2020).

24 <https://www.nice.org.uk/guidance/NG159>; other rapid guidelines for patients with systemic anticancer treatments and dialysis service delivery have also been introduced.

25 See, generally, J Harris , “The Age-Indifference Principle and Equality” 14 (2005) Cambridge Quarterly of Healthcare Ethics 93 1574748610.1017/s0963180105050103.

26

A Extance , “Covid-19 and Long Term Conditions: What If You Have Cancer, Diabetes, or Chronic Kidney Disease?” (2020) BMJ 10.1136/bmj.m117432213482 <https://www.bmj.com/content/368/bmj.m1174> (last accessed 26 March 2020).

27

S Bosley , “Testing for Coronavirus: What Is Being Done in the UK?” (2020) The Guardian <https://www.theguardian.com/world/2020/mar/17/testing-for-coronavirus-what-being-done-in-uk> (last accessed 23 March 2020).

28
ibid.

29

A Rimmer , “Covid-19: GPs Can Stop Health Checks for Over 75s and Routine Medicine Reviews” (2020) BMJ 10.1136/bmj.m115732198147 <https://www.bmj.com/content/368/bmj.m1157> (last accessed 23 March 2020).

30 Coronavirus Act 2020.

31 “Coronavirus: Testing Rolled Out for Frontline NHS Staff” <https://www.bbc.co.uk/news/uk-52072568> (last accessed 30 march 2020).

32 Bosley, supra, note 27.

33

D Sokol , “The Life and Death Decisions of Covid-19” (2020) BMJ <https://blogs.bmj.com/bmj/2020/03/20/daniel-sokol-the-life-and-death-decisions-of-covid-19> (last accessed 24 March 2020).

34

SH Ebrahim , QA Ahmed , E Gozzer , P Schlagenhauf and ZA Memish , “Covid-19 and Community Mitigation Strategies in a Pandemic” (2020) BMJ 10.1136/bmj.m106632184233 <https://www.bmj.com/content/368/bmj.m1066> (last accessed 31 March 2020).

35 Germain, supra, note 5, 39–40.

36 Resnick, Galea and Sivashanker, supra, note 18.

37
ibid.

38

R Clarke , “We Can’t Be Squeamish about Death. We Need to Confront Our Worst Fears” (2020) The Guardian <https://www.theguardian.com/world/2020/mar/14/talk-about-death-coronavirus-covid-19-nhs-doctors-patients> (last accessed 24 March 2020).

39

J Parker and M Mirzaali , “The Moral Cost of Coronavirus” (2020) Journal of Medical Ethics <https://blogs.bmj.com/medical-ethics/2020/03/16/the-moral-cost-of-coronavirus> (last accessed 23 March 2020).

40 At the clinical level, bioethics philosophers generally believe that the healthcare decision-making process should revolve around four principles: autonomy, non-maleficence, beneficence and justice. In line with these principles, doctors select treatments based on an assessment of the disease and only then run a cost–benefit analysis for each treatment option. TL Beauchamp and JF Childress , Principles of Biomedical Ethics (Oxford, Oxford University Press 2001 ) p 12.

41
ibid.

42

G Morley , “COVID-19 and the Moral Community: A Nursing Ethics Perspective” (2020) Journal of Medical Ethics <https://blogs.bmj.com/medical-ethics/2020/03/25/covid-19-and-the-moral-community-a-nursing-ethics-perspective> (last accessed 26 March 2020).

43

O Goldhill , “Ethicists Agree on Who Gets Treated First When Hospitals Are Overwhelmed by Coronavirus” (2020) Quartz <https://qz.com/1821843/ethicists-agree-on-who-should-get-treated-first-for-coronavirus> (last accessed 27 March 2020).

44

S Berinato , “That Discomfort You’re Feeling Is Grief” (2020) Harvard Business Review <https://hbr.org/2020/03/that-discomfort-youre-feeling-is-grief> (last accessed 30 March 2020).

